# Prognosis of clinical pneumonia in undernourished children in rural Gambia

**DOI:** 10.1186/s41479-025-00194-8

**Published:** 2026-03-25

**Authors:** Yasir Shitu Isa, Megan Carelus, Isabelle Silber, Rasheed Salaudeen, Golam Sarwar, Yekini Ajao Olatunji, Ilias Hossain, Isaac Osei, Galega Lobga, Banjo Adeshola, Ousman Barjo, Momodou M. Drammeh, Umberto D’Alessandro, Patricia L. Hibberd, Grant A. Mackenzie, Clarissa Valim

**Affiliations:** 1 Medical Research Council Unit, The Gambia, London School of Hygiene & Tropical Medicine, Fajara, The Gambia; 2 Boston University School of Public Health, Boston, MA USA; 3 Faculty of Infectious and Tropical Diseases, London School of Hygiene & Tropical Medicine, London, UK; 4 Department of Global Health, Boston University School of Public Health, Boston, MA USA

## Abstract

**Background:**

Undernutrition significantly increases the risk of severe infections and mortality in children under five, particularly in low- and middle-income countries. Pneumonia, a leading cause of childhood death, is especially dangerous in undernourished children, yet prognostic measures to identify those at highest risk are lacking.

**Objective:**

To identify algorithms of poor prognosis in undernourished children with clinical pneumonia for early identification of children at risk for poor outcomes.

**Methods:**

This study analyzed a subset of children enrolled in a cohort designed to identify biomarkers of bacterial pneumonia. Children aged 2–59 months with clinical pneumonia were recruited from two rural Gambian hospitals. Clinical and anthropometric data were collected at baseline, during hospitalization, and at 30-day follow-up. Nutritional status was classified using WHO definitions for stunting (height-for-age Z-scores) and wasting (weight-for-height Z-scores) as severe (Z-scores ≤ -3), moderate (-2 ≥ Z-scores > -3), and mild (-1 ≥ Z-scores > -2). Prognostic outcomes were classified into good and poor. Poor prognosis included death, prolonged hospital stay (≥ 7 days), post-discharge care-seeking, and difficult to feed during admission. Good prognosis was based on a hospital stay < 3 days, with good outcomes within 30 days of the initial visit. Classification tree models and penalized logistic regression models (fit through elastic net) were used to identify combinations of predictors of poor prognosis (prognostic signatures).

**Results:**

A total of 246 children with clinical pneumonia and undernutrition (wasting or stunting) were included. Children with poor prognosis presented more frequently with respiratory distress, hypoxemia, reduced capacity oforal feeding difficulty, and anemia. As expected, undernutrition was associated with adverse outcomes. The final prognostic algorithms were accurate to identify undernourished children at risk of poor prognosis: with sensitivity and specificity > 80% and area under the receiver operating characteristic curve ≥ 0.80. Furthermore, we identified accurate prognostic signatures among children with both wasting and stunting.

**Conclusion:**

Measures collected at admission in undernourished children with clinical pneumonia can identify those at risk of poor outcomes. The prognostic signatures developed in this study may inform early risk stratification and guide timely intervention, particularly in resource-limited settings.

**Supplementary Information:**

The online version contains supplementary material available at 10.1186/s41479-025-00194-8.

## Introduction

Malnutrition is defined by the World Health Organization (WHO) as a deficiency, excess or imbalance in energy or nutrient intake [1]. It includes both undernutrition and overnutrition; undernutrition encompasses wasting, stunting, underweight, and micronutrient deficiencies [2]. Undernutrition is the most common form of malnutrition among children under five in low- and middle-income countries (LMICs) and although lower-middle-income countries are home to about half of the world’s under-five population, they account for roughly 60% of children with stunting and around 75% of children with wasting [3]. Furthermore, 45% of deaths in children under five years of age, particularly in LMICs, are attributed to undernutrition [4].

In 2020, an estimated 150.2 million and 42.8 million children under 5 years of age were affected globally by stunting and wasting, respectively [3]. Stunting, defined by low height-for-age z scores (HAZ), is associated with chronic undernutrition, whereas wasting, characterized by low weight-for-height z scores (WHZ), is associated with acute undernutrition [5]. On the basis of the WHO Z score threshold levels, stunting and wasting are categorized as mild, moderate, or severe [6, 7]. Children who are undernourished and exhibit either wasting or stunting face an increased risk of infectious diseases and are more likely to experience severe illness and death than their well-nourished counterparts [8]. Several studies have established that deaths are due to increased susceptibility to life-threatening infections, with diarrhoea and pneumonia being the most common [9, 10].

Undernourished children with clinical pneumonia tend to have poor outcomes. Acute infections worsen nutritional status, leading to a vicious cycle of infections and undernutrition, and thus contribute to poorer outcomes [8]. Studies have shown that children with pneumonia and stunting or wasting have longer durations of hospital stay and higher rates of treatment failure and overall mortality [11–13]. A study conducted in Bangladesh revealed that children with severe acute malnutrition (defined as having WHZ − 3 or MUAC > 11.5 cm or bilateral pitting edema) and danger signs of severe pneumonia had significantly higher rates of treatment failure infections (defined as requiring change in antibiotics) (58% vs. 20%; *p* < 0.001) and mortality (21% vs. 4%; *p* < 0.001) than did those without danger signs [14]. A cohort study in Kenya revealed that 25% of children admitted with severe pneumonia were undernourished, and 37% of these children died after discharge [15]. In The Gambia, among 3,630 children admitted with suspected meningitis, sepsis, and pneumonia, the risk of death in children with clinical pneumonia and clinically severe malnutrition was 8 times (95% CI = 4–15) greater than that in those without clinically severe malnutrition [16]. Clinically severe malnutrition was defined as visible wasting of the buttocks, characteristic skin or hair changes, or bilateral pedal oedema.

Pneumonia may present with less clear signs of respiratory distress in undernourished children, leading to underdiagnosis, delayed diagnosis and suboptimal care [13]. Furthermore, the uncharacteristic clinical presentation makes it challenging to assess the severity of pneumonia in these children and establish appropriate clinical management. Therefore, an algorithm to predict poor prognosis among undernourished children with clinical pneumonia could support clinical care and reduce the mortality risk in areas where healthcare resources are limited. In Gambian children who were undernourished and had clinical pneumonia, we sought to identify a combination of factors, so called prognostic signature, that could predict poor prognosis.

## Methods

We included a subset of children originally enrolled in a prospective observational study seeking to detect biomarkers for bacterial pneumonia among children with clinical pneumonia [17]. Clinical information was collected by research nurses and paediatricians from children diagnosed with clinical pneumonia at the time of presentation, including signs and symptoms associated with the respiratory, digestive, and nervous tract, in addition to other signs of infection such as skin infections. Further, feeding ability was assessed daily for seven days during their hospital admission, if hospitalized. Although discharge decisions were done by government nurses, hospital admitted children were evaluated at discharge and classified as discharged well or not, with the first defined based on attaining clinical stability with normal oral feeding and improved respiratory symptoms. Thirty (30) days after the initial encounter, children were called to assess whether they were alive, and if so, whether they had sought outpatient care for their original respiratory symptoms, or whether they were re-admitted. Participants were recruited from the Basse District Hospital and Bansang Hospital in rural Gambia [17].

### Inclusion and exclusion criteria

The parent study enrolled all children aged 2–59 months with clinical pneumonia, defined as cough and/or difficulty breathing, and at least one of the following signs were eligible: lower chest wall indrawing, increased respiratory rate for age according to WHO criteria, arterial oxygen saturation < 93%, grunting, nasal flaring or undernutrition [17]. A clinician assessed the need of each patient for hospital admission. Children admitted to a hospital in the previous 2 weeks, suspected of having tuberculosis on the basis of a > 2-week history of cough, or who had HIV infection were excluded [17]. This analysis included all children from the original cohort with HAZ or WHZ ≤ -1 and who met the prognosis criteria detailed below.

### Patient management

All admitted children were managed according to WHO guidelines for paediatric pneumonia, which include parenteral ampicillin and gentamicin, oral or nasogastric feeding, and monitoring of clinical signs of respiratory distress [18]. Those with other conditions, such as sepsis, meningitis and other comorbidities, received treatment following hospital guidelines. Intranasal oxygen, blood transfusion, and other supportive care were provided as necessary. Children managed as outpatients received standard of care with oral antibiotics and were followed through a phone call 30 days within the first contact.

### Study measurements

For all enrolled participants, a drop of capillary blood was collected through finger prick for a rapid malaria diagnostic test (WHO- prequalified mRDT), and a rapid hemoglobin test (Hemocue, etc.). Additionally, 5 mL of blood was collected for laboratory analysis: for bacterial culture, full blood cell count (FBC), malaria microscopy (thick and thin blood smears), as well as storage of plasma in a biobank. A chest X-ray was conducted for radiological abnormalities.

### Undernutrition classification

Undernutrition included wasting or stunting. We used the WHO classification of undernutrition with wasting defined based on WHZ as severe (WHZ ≤ -3), moderate (-3 < WHZ ≤ -2) and mild (-2 < WHZ ≤-1). Stunting was defined based on HAZ as severe (HAZ ≤ -3), moderate (-3 < HAZ ≤ -2) and mild (-2 < HAZ ≤ -1) [19]. Additionally, children with moderate or severe stunting and/or wasting were classified as having moderate or severe undernutrition, whereas those with mild stunting and/or mild wasting were classified as having mild undernutrition.

### Outcome classification

A good prognosis was assigned to children who had a hospital stay < 3 days, were not admitted or were discharged well, did not experience reduced ability to maintain oral intake during admission and also, within 30 days from the first encounter did not: seek outpatient care for the original respiratory symptoms; undergo a hospital readmission; did not die. (Fig. S1). Children were categorized as discharged well when they were clinically stable, with normal oral feeding and had improved respiratory symptoms. Poor prognosis was assigned to children who died either in the hospital or within the 30-day follow-up period as well as those who were discharged well and alive at the 30-day follow-up and met any of the following conditions: had a hospital stay ≥ 7 days; sought outpatient care or were re-admitted after discharge; or had a reduced ability to maintain oral feed during hospitalization.

### Ethics

Written informed consent was obtained from the parents or legal guardians of the children. The study was approved by the Gambia Government/MRCG Joint Ethics Committee and the institutional review board of Boston University Medical Centre.

### Statistical analysis

Baseline comparisons of demographic and clinical characteristics between children with good and poor prognosis as well as children with different levels of undernutrition were based on t-tests or Wilcoxon rank sum tests when continuous characteristics were analysed, and chi-square tests or Fisher’s exact tests when categorical characteristics were analysed. To verify in our cohort the increased chances of poor outcomes of children with increased severity of undernutrition, we estimated ORs for the association of poor (vs. good) prognosis with mild (vs. moderate or severe undernutrition) through logistic regression models. In these models, considering that clinical symptoms were expected to be highly correlated with undernutrition, estimates were only adjusted by the commonly used demographic parameters: sex and age.

Prognostic signatures were sought through classification trees (CART) and penalized logistic regression models fit through elastic nets to allow modelling correlated predictors. All children with undernutrition were included in these analyses. Model outcomes were poor vs. good prognosis and candidate predictors included demographic characteristics (age and sex), chest X-ray findings (presence of consolidation), and clinical parameters present at admission in fifteen children or more (see Table 1), such as age, sex, partial oxygen saturation (SpO_2_) level, diarrhoea, respiratory rate, measured temperature, vomiting, lethargy, anaemia and haemoglobin levels obtained in a rapid haemoglobin test. All these parameters have been found in previous studies to predict outcomes of clinical pneumonia. Candidate predictors were those allowed to be selected by signatures. Z-scores were also included in the pool of candidate predictors in some models because they could be surrogates of the severity of undernutrition. However, because Z-scores may be difficult to calculate by nurses at admission, some signatures excluded Z-scores from the pool of candidate predictors. Laboratory parameters obtained through a full blood cell count (FBC) might help to determine the severity of the inflammatory process and may support decisions about the aetiology of the clinical pneumonia but they may not be available in LMIC health services. Therefore, signatures with and without FBC parameters among candidate predictors were sought. FBC parameters included white blood cell count, lymphocyte percentage and count, and neutrophil percentage and count. To develop an effective screening algorithm tool with higher sensitivity than specificity, we focused on fitting trees by penalizing misclassification of a child with a true poor prognosis twice as heavily as misclassification of a child with a true good prognosis. Accuracy of identified signatures was estimated based on sensitivity, specificity, overall accuracy (proportion of children correctly classified) and area under the receiver operating characteristic curve (AUC-ROC) with corresponding 95% CIs. DeLong’s method was used to estimate 95% Cis of AUC-ROCs.

Trees were split via the Gini index and were pruned through cross-validation. Tunning parameters for penalized regression were selected through 5-fold cross-validation given an alpha of 0.9 that was chosen to be the highest that allowed correlation among predictors. Missing data on candidate predictors were imputed through the automated tree algorithm based on surrogate predictors. Penalized logistic regression was based on complete data and excluded children missing FBC counts to allow comparisons between models with and without FBC parameters among candidate predictors.

All analysis was also stratified by specific type of undernutrition, i.e., wasting and stunting. Analyses was conducted using R v9.4 with the addition of the following packages: Rpart version 4.1.19 [20], glmnet version 4.1-8 [21], pROC version 1.18.4 [22], caret [23], and epitools version 0.5–10.1 [24] Z-scores were estimated based on STATA tables and calculations.

## Results

### Study population and undernutrition

**Fig. 1:**
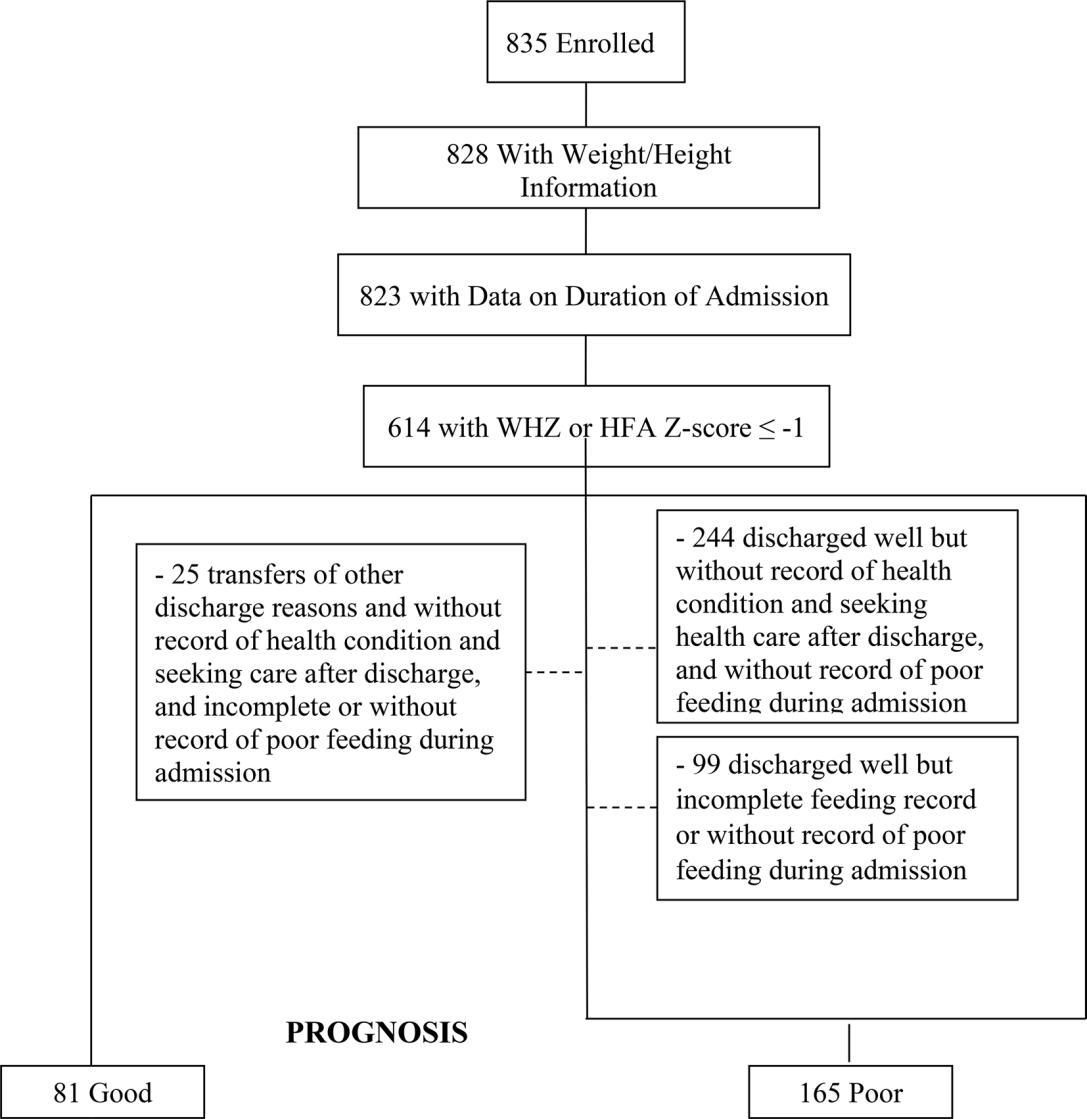
Selection of the subset of children included in this analysis from the 835 enrolled in the parent study

**Fig. 2:**
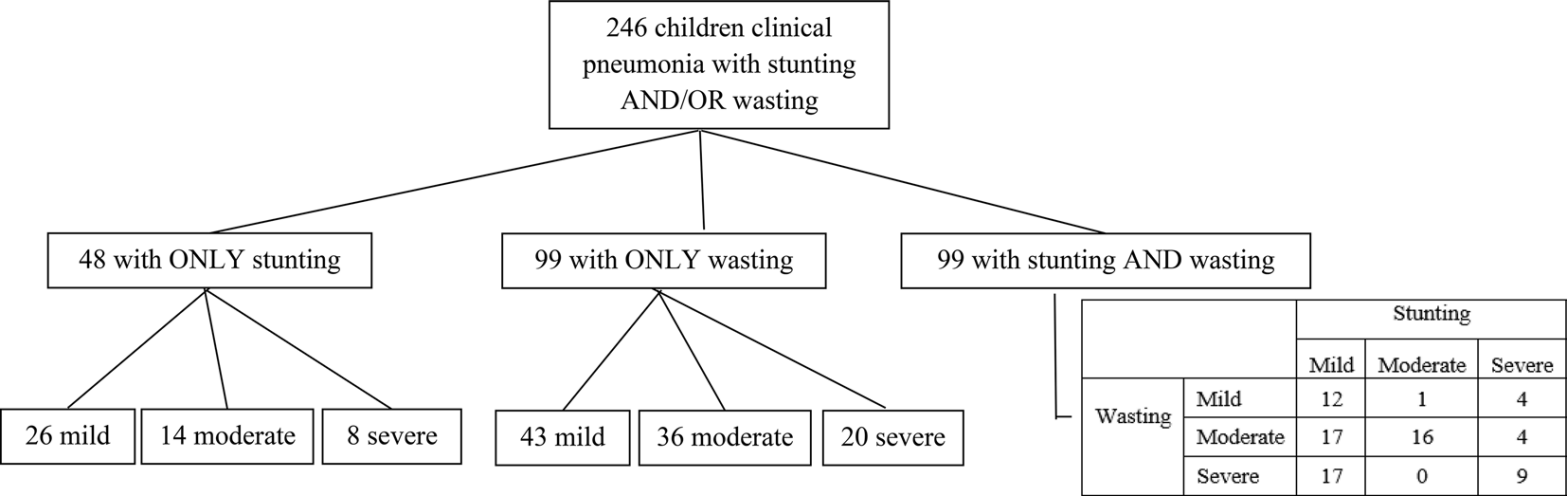
Number of enrolled children with undernutrition (stunting and/or wasting)

A total of 246 children had information about undernutrition, WHZ or HAZ ≤ -1 and met one of our two prognosis criteria (Fig. 1); 81 children had a good prognosis and 165 had a poor prognosis. Among these children, 48 (20%) had only stunting, 99 (40%) had only wasting, while 99 (40%) had a combination of stunting and wasting (Fig. 2). Of those with wasting only, 43 (43%) had mild, 36 (36%) had moderate, and 20 (20%) had severe wasting. Among those with stunting only, 26 (54%) had mild, 14 (29%) had moderate, and 8 (17%) had severe stunting. Among children with both stunting and wasting, 29 (29%) had moderate or severe stunting and wasting.

Compared with children who had mild undernutrition, those with moderate to severe undernutrition were more prone to lethargy and diarrhoea (Table S1). They also tended to have higher rates of anaemia and blood-culture–confirmed bacterial infections, although these differences were not statistically significant.

### Prognosis of clinical pneumonia

As expected, children with poor prognosis, when compared with those with a good prognosis, were younger, had lower oxygen saturation levels, were more likely to be lethargic, and to present with diarrhoea and vomiting (Table 1). Furthermore, consolidation was more frequently observed among children with a poor than a good prognosis, possibly indicating a bacterial aetiology. Anaemia was also more frequent among children with a poor than a good prognosis, although differences in both haemoglobin measured through the rapid test and through haematocrit were not statistically significant. Malarial co-infections and bacterial infections confirmed through blood culture were more common in children with a poor prognosis, although they were infrequent in both groups and differences were not statistically significant. Consistent with previous work, as severity of undernutrition increased, the odds of a poor prognosis when comparing to a good prognosis increased in crude and adjusted analysis (*P* < 0.0001), with children with severe undernutrition in analysis adjusted for sex and age presenting with an odds of poor prognosis 4.67 (95% CI = 2.24, 10.29) larger than those with mild undernutrition (Table 2). Overall, only 15 children died during or within 30 days from admission; 1 of them with mild undernutrition, 3 with moderate and 11 with severe undernutrition.

Differences between children with poor and good prognosis of among those with wasting (Table S2) and with stunting (Table S3) were comparable to differences among those with any form of undernutrition. Of note, the proportion of children with poor prognosis increased with the severity of wasting and stunting (Fig. S2). Among children with mild wasting, 47% had poor prognosis while this increases to 70% in those with severe wasting. In those with mild stunting, 56% had poor prognosis compared to 88% with severe stunting. Children with both moderate-severe wasting and stunting had higher proportion of poor prognosis at 84% compared to 64% in those with mild wasting and stunting. Further, the increased odds of a poor prognosis in children with increase in severity of undernutrition was also observed when analysing separately children with wasting (*P* = 0.002 in crude analysis and 0.003 in adjusted analysis) and with stunting (*P* = 0.04 in crude analysis and 0.048 in adjusted analysis) (Table 2).

### Prognostic signatures of clinical pneumonia

**Fig. 3:**
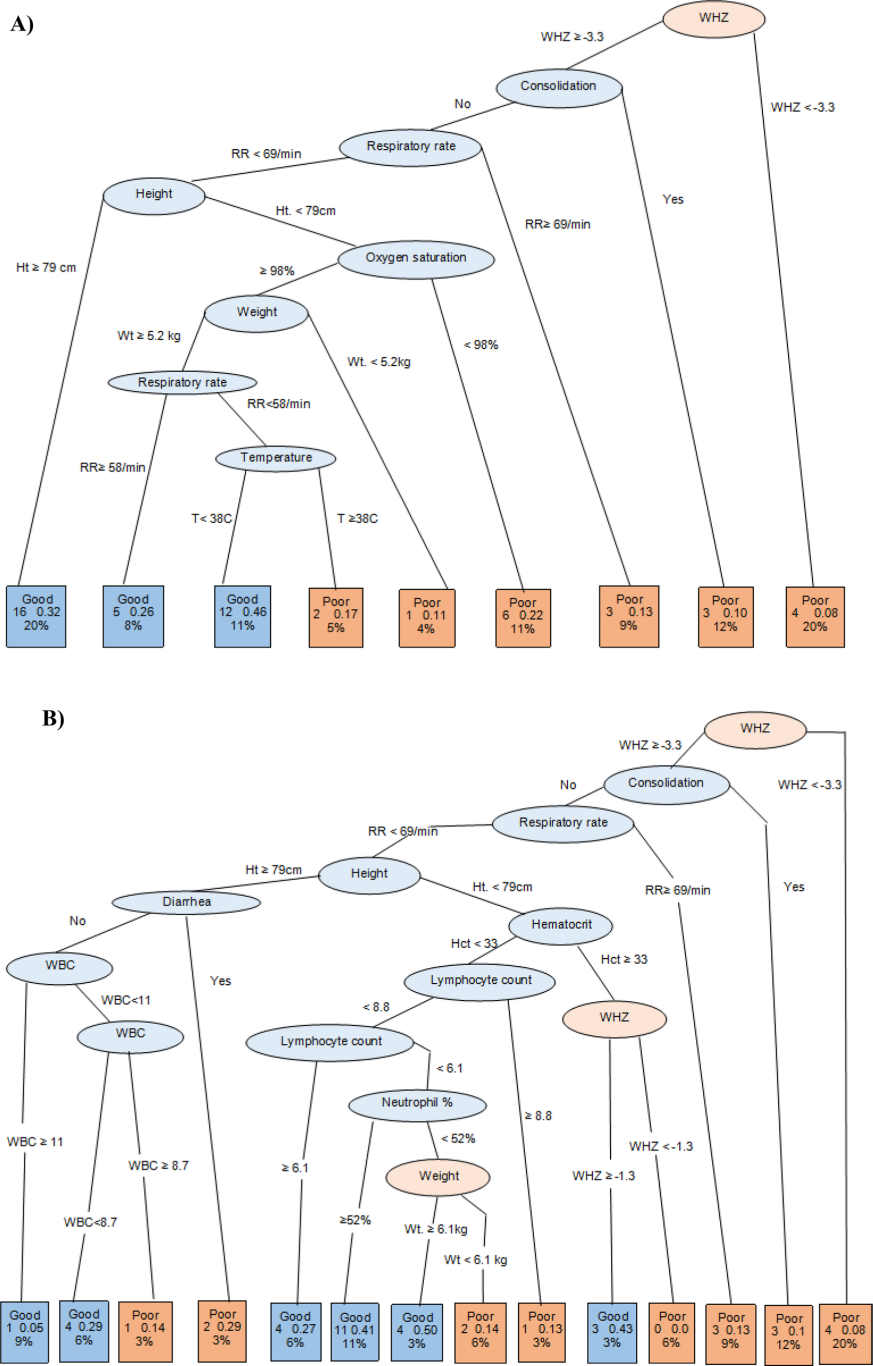
Prognostic signature among undernourished children (weight-for-height Z-scores [WHZ] or height-for-age Z-scores [HAZ] < -1) obtained in classification trees including (**A**) and excluding (**B**) parameters from a full blood cell count among candidate predictors. Ellipses contain name of variables discriminating poor from good prognosis in intermediate nodes. Terminal nodes (squares) include the predicted prognosis of children, the number (and percent %) of children in the node, as well as the proportion of children in each node with a poor prognosis. O_2_ Sat = partial oxygen saturation; WBC = white blood cell count

Using classification trees, we identified two prognostic signatures for poor clinical pneumonia prognosis (vs. good prognosis) among children with WHZ or HAZ ≤ 0. In the first signature (Fig. 3A), candidate predictors excluded parameters of the full blood count (FBC) because it is not always readily available in LMIC health services. In the second signature, FBC parameters were included among candidate predictors (Fig. 3B). In both signatures children with WHZ < -3.3 were predicted to have a poor prognosis, as well as those with WHZ ≥ -3.3 who also had consolidation in the chest X-ray. Also, in both signatures, children with WHZ ≥ -3.3 and without consolidation in the chest X-ray but with elevated respiratory rate (≥ 69/min) were predicted to have a poor prognosis. Differences between these signatures were observed among children with WHZ ≥ -3.3, without consolidation in the chest X-ray, and with respiratory rate < 69/minute: while the first tree predicted poor prognosis based on weight, height, SpO_2_ and temperature, the second tree used further information on lymphocyte count, neutrophil percent, and white blood cell count in addition to a combination of weight, height and diarrhoea. Both signatures attained a prognostic sensitivity larger than 80% (Table 3). The specificity and AUC-ROC of the prognostic signature without FBC parameters was 80% (95% CI = 70%, 88%) and 0.89 (95% CI = 0.82, 0.91), respectively, and the second signature specificity and AUC-ROC was 77% (95% CI = 66%, 85%) and 0.82 (95% CI = 0.73, 0.84), respectively.

In the second signature, after excluding the Z-scores, the tree-based models generally selected the same clinical parameters (Fig. S3), although lymphocyte percent from FBC was included when FBC parameters were included among candidate predictors. Although the sensitivity of the tree-based signature that excluded FBC parameters from candidate predictors was higher (88%) than the signature that included FBC parameters (82%), specificity was lower: 68% when FBC parameters were excluded versus 83% when FBC parameters were included (Table S4). When conducting separate analysis for children with wasting (Fig. S4) and stunting (Fig. S5), prognostic signatures based on classification trees that included and excluded Z-scores and parameters had comparable and high accuracy (Table S5), with sensitivity ranging from 81% to 92%, specificity ranging from 74% to 87%, and AUC-ROCs varying from 0.81 to 0.89.

Additional prognostic signatures were identified through penalized regression in all undernourished children (Tables 4 and S6) and when analysing children with wasting (Table S7) and stunting (Table S8) separately. However, those signatures attained poor specificity (ranging from 35% to 57%) (Tables 3 and S5).

## Discussion

We identified accurate signatures to prognosticate clinical pneumonia in undernourished children, i.e., those with wasting or stunting. The first signature with highest sensitivity and specificity included measurement of WHZ, presence of consolidation, respiratory rate count, and measurements of weight, height and temperature. Our second signature attained comparable accuracy and excluded Z-scores but included haematocrit, lymphocyte count from a full blood cell count in addition to weight, age, partial oxygen saturation measurement, respiratory rate count, and presence of diarrhoea and consolidation. Other accurate signatures were identified when including only children with wasting and stunting in analysis that could be deployed in areas where one specific type of undernutrition is predominant. However, all signatures obtained via penalized logistic regressions had poor specificity, although excellent sensitivity, resulting in a less effective method even for screening patients at high risk of poor prognosis to support care.

Identifying prognostic signatures for undernourished children with clinical pneumonia, is important because warning signs of clinical pneumonia may be absent in these children and they are under high risk of poor outcomes. Consistent with previous work, although our sample size yield unprecise estimates with wide confidence intervals, we showed that the odds of poor outcomes in children with poor prognosis increases as severity of undernutrition increases. These findings are consistent with previous studies in LMICs that reported malnutrition as a major driver of inpatient and post discharge mortality in patients with pneumonia [15, 16, 25].

A previous study conducted in India identified factors such as delayed hospital referral, incomplete immunization, severe malnutrition, refusal to feed, and hypoglycaemia as contributors to poor prognosis [26]. However, the study focused solely on mortality as the outcome measure. In a case‒control study conducted in Bangladesh, Chisti et al. identified hypoxemia as a key predictor of morbidity and mortality among children with clinical pneumonia and severe acute malnutrition [27]. The study highlights that limited access to pulse oximetry in resource-constrained settings impedes the early identification of high-risk children who require optimized care. This emphasizes the importance of identifying alternative, easily measurable predictors of poor outcomes to help reduce morbidity and mortality in this vulnerable population.

Our study adds to the existing body of knowledge by identifying additional, combinations of markers to prognosticate outcomes of clinical pneumonia in low-resource settings in paediatric patients with undernutrition. Furthermore, we broadened the definition of poor prognosis beyond mortality to include other clinically significant outcomes that may help in identifying children who are more vulnerable and in greater need of prioritized care. There were limitations in this analysis. First, owing to the limited sample size, we could not split the data to have a separate dataset to obtain estimates of accuracy. Therefore, our estimates of accuracy of the identified signatures may be overestimated. Another limitation is the lack of follow-up information for several children from the original study. Children whose follow-up status (alive/dead, feeding status during follow-up or seeking care after discharge) was missing and did not meet criteria for moderate prognosis based on duration of admission nor decreased feeding during admission could not be assigned to a prognosis group, and were therefore excluded from this analysis, limiting our sample size and potentially introducing biases. Additionally, clinical evaluations are always subject to misclassification errors and may be specific to a certain team of clinicians, which limits the generalizability of the findings. The clinical team involved in evaluating these participants was large and included several well-trained nurses and clinicians, suggesting that our results would be generalizable to other clinical teams. For all reasons listed above, it will be important to assess the reproducibility of our results with different clinical teams working in different settings.

Additionally, it would be important to compare the risk of death among undernourished children with moderate to severe stunting and wasting. We only observed 15 deaths preventing precise analysis including death as an outcome.

In conclusion, we identified algorithms based on simple predictors that could be implemented on a mobile device, thereby assisting healthcare workers in prioritizing care for children presenting with clinical pneumonia. For areas with predominance of stunting or wasting, we identified prognostic algorithms focused on the specific type of undernutrition. The use of such predictors may improve outcomes and reduce mortality associated with undernutrition in patients with clinical pneumonia.

### Future directions

Future research directions include validating the proposed prognostic algorithms in a larger number of undernourished children from different geographical areas. This is particularly important considering that classification trees are a nonparametric method, and the cut-off values of continuous clinical predictors may not be precise.

## Conclusion

Undernutrition, especially wasting, is a key predictor of poor outcomes in children with clinical pneumonia. Our study expands on existing knowledge by identifying simple, easy-to-measure clinical and laboratory markers that predict poor prognosis beyond mortality. The incorporation of additional biomarkers may further increase the accuracy of identifying high-risk children. We developed three predictive algorithms to assist healthcare workers in low-resource settings in prioritizing care for vulnerable children. Early detection of these factors may help stratify high-risk children to receive better care, thereby reducing mortality and morbidity. Our findings indicate that algorithms such as our first signature, which combines predictors including weight, height, respiratory rate, temperature, oxygen saturation, and radiographic evidence of consolidation could be implemented in an application to help detect early signs of poor prognosis. Despite limitations such as a small sample size and loss to follow-up, these tools have the potential to improve clinical decision-making and outcomes. Future validation in larger and diverse populations is essential to confirm their effectiveness and generalizability.

**Table 1:** Participants characteristics by prognosis status

Participant Characteristic	Prognosis		
	Good (*N* = 81)	Poor (*N* = 165)	*P*†
*Demographic*			
Age (months), median (IQR)	15 (9, 27)	12 (6, 20)	0.01
Sex (female), n (%)	39 (48%)	72 (44%)	0.50
**Clinical History and Examination on Admission**			
Oxygen saturation (%), mean ± SD	98.0 ± 2.4	96.4 ± 5.5	0.002
Respiratory rate,/min, median (IQR)	53 (48, 61)	55 (46, 64)	0.12
Axilliary Temp, °C, mean ± SD	37.6 ± 1.2	37.7 ± 1.2	0.77
Antibiotic use (within past week), n (%)	15 (20%)	35 (26%)	0.62
Lethargy n, (%)	11 (14%)	50 (30%)	0.004
Unconsciousness, n (%)	0 (0)	2 (1.2%)	0.55*
Convulsions, n (%)	2 (2.5%)	7 (4.2%)	0.72*
Neck stiffness, n (%)	0 (0%)	2 (1.2%)	1*
Bulging fontanel, n (%)	0 (0)	1 (0.6)	1*
Diarrhoea, n (%)	16 (20%)	56 (34%)	0.02
Vomiting, n (%)	3 (3.7%)	24 (15%)	0.01*
**X-ray Results**			
Other infiltrates, n (%)	1 (1.4%)	8 (5%)	0.28*
Consolidation, n (%)	3 (4%)	32 (21%)	0.0004*
Pleural Effusion, n (%)	1 (1.4)	1 (0.7)	1*
**Undernutrition and Anemia**			
Weight-for-height Z-score, mean ± SD	-1.63 ± 1.31	-2.34 ± 1.92	0.0008
Height-for-age Z-score, mean ± SD	-0.73 ± 1.51	-1.37 ± 1.65	0.0028
Hemoglobin, mean ± SD	10.6 ± 1.7	9.7 ± 2.1	0.14
Anemia, n (%)			0.005*
Mild	26 (32%)	36 (22%)	
Moderate	18 (43%)	72 (44%)	
None	34 (42%)	48 (29%)	
Severe	2 (0.8%)	7 (4%)	
**Infection and Inflammation Associated Laboratory Markers**			
WBC count (10^9^/L) mean ± SD	13.8 ± 7	15.1 ± 9	0.24
Neutrophil count (10^9^/L), median (IQR)	5.6 (3.4, 10.1)	6.4 (4.0, 10.0)	0.49
Neutrophil %, median (IQR)	50 (38, 65)	52 (37, 70)	0.69
Lymphocyte count (10^9^/L, median (IQR)	4.66 (3.05, 6.37)	4.60 (3.18, 6.37)	0.76
Lymphocyte %, median (IQR)	39 (24, 53)	38 (25, 52)	0.81
Hematocrit g/dL median (IQR)	30 (28,33)	29 (26,32)	0.09
Invasive Bacterial Infection, n (%)	2 (2.5)	11 (6.7)	0.23
Malarial Infection*, n (%)	2 (2.5)	7 (4.4)	0.72

SD = standard deviation; WBC = white blood cells

†P values were estimated with t tests when reporting means, Wilcoxon tests when reporting medians, and Pearson chi-square tests when reporting proportions except where marked * when Fisher exact tests were used

**Table 2:** Crude and adjusted odds ratios (ORs) and 95% confidence intervals (CIs) for the associations between poor prognosis and undernutrition (wasting or stunting), wasting, and stunting. Estimates were obtained in logistic regression models and p-values obtained through likelihood ratio tests and the reference categories are marked with (-)

Exposure	*N*†	No. Poor Prognosis (%=*n*/NŦ)	Crude OR (95% CI)	*P*	Adjusted OR* (95% CI)	*P*
**Undernutrition**				< 0.0001		< 0.0001
Mild Undernutrition	83	44 (53%)	–		–	
Moderate undernutrition	83	53 (64%)	1.57 (0.84, 2.93)		1.38 (0.73, 2.62)	
Severe Undernutrition	80	68 (85%)	5.0 (2.43, 11.00)		4.67 (2.24, 10.29)	
Sex, Male	135	93 (69%)		0.50	–	0.66
Female	111	72 (65%)	0.83 (0.49, 1.42)		0.88 (0.50, 1.66)	
Age (months)	246	165 (67%)	0.97 (0.95, 0.99)	0.009	0.98 (0.95, 0.997)	0.03
**Wasting**				0.002		0.003
Mild Wasting	70	40 (57%)	–		–	
Moderate Wasting	65	43 (66%)	1.47 (0.73, 2.97)		1.28 (0.62, 2.64)	
Severe Wasting	63	53 (84%0	3.98 (1.79, 9.44)		3.84 (1.70, 9.29)	
Sex, Male	105	75 (71%)	*	*	–	0.47
Female	93	61 (66%)	*	*	0.79 (0.42, 1.50)	
Age (months)	198	62	*	*	0.96 (0.42, 1.50)	0.005
**Stunting**				0.04		0.048
Mild Stunting	75	53 (71%)	–		–	
Moderate Stunting	46	32 (70%)	0.95 (0.43, 2.14)		0.88 (0.39, 2.03)	
Severe Stunting	26	24 (92%)	4.98 (1.32, 32.7)		4.64 (1.20, 30.81)	
Sex, Male	87	62 (71%)	*	*	–	0.23
Female	60	47 (78%)	*	*	1.63 (0.74, 3.62)	
Age (months)	147	109 (74%)	*	*	0.97 (0.94, 1.00)	0.06

^**†**^The sample size included in each analysis varied because in “Undernutrition”, children could have HAZ or WHZ ≤ -1 but not both and be included in analysis. However, analysis of wasting only included children with WHZ ≤ -1 and analysis of stunting only included children with HAZ ≤ -1

^**Ŧ**^The proportion in this column represents the number of children with poor prognosis divided by the total number of children within the same category

*Estimates and P-values for age and sex in all bivariate models were comparable to those in the Undernutrition section of the table, although the sample size included in the analysis had some slight variation

**Table 3:** Accuracy of prognostic signatures identified through classification tree and logistic penalized regression (fit through elastic net; ENET): sensitivity, specificity, overall accuracy, and area under the receiver operating characteristic curve (AUC-ROC), all with corresponding 95% confidence intervals (CIs)

	Sensitivity (95% CI)	Specificity (95% CI)	Overall Accuracy (95% CI)	AUC-ROC
	All children with Undernutrition			
Tree with Clinical Predictors and Z-scores	84% (77%, 89%)	80% (70%, 88%)	0.83 (0.77, 0.87)	0.87 (0.82, 0.91)
Tree with Clinical, Z-scores and FBC parameters	80% (73%, 86%)	77% (66%, 85%)	0.79 (0.73, 0.84)	0.82 (0.76, 0.88)
ENET with Clinical Predictors and Z-scores	84% (76%, 90%)	45% (32%, 60%)	0.71 (0.63, 0.78)	0.75 (0.67, 0.83)
ENET with Clinical predictors, Z-scores and FBC parameters	82% (73%, 89%)	57% (42%, 70%)	0.74 (0.66, 0.80)	0.75 (0.68, 0.83)

FBC = full blood cell count

*P-value of the AUC-ROC estimated through DeLong’s method

**Table 4:** Prognostic signature for 246 children with mild to severe undernutrition (weight-for-height Z-scores [WHZ] or height-for-age Z-scores [HAZ] ≤ -1), comparing those with poor (*N* = 165 children) and good (*N* = 81) prognoses estimated using a penalized logistic regression model (fit through elastic net). Children without full blood cell count were excluded from this analysis

Predictors	Odds Ratios
Not Including FBC Parameters	
Intercept	1.95
Diarrhoea (yes)	1.0
Vomit (yes)	1.03
Consolidation in the chest X-ray	1.98
Weight (Kg)	0.92
Height-for-Age Z-scores	0.96
**Including FBC Parameters**	
Intercept	2.04
Lethargy (yes)	1.02
Diarrhoea (yes)	1.11
Vomit (yes)	1.09
Consolidation in the chest X-ray	2.17
Weight (Kg)	0.91
Height-for-Age Z-scores	0.95

FBC =  full blood cell count

## Supplementary Information

Below is the link to the electronic supplementary material.

Supplementary Material 1

## Data Availability

No datasets were generated or analysed during the current study.
