# Supplementary material for: Prognosis of clinical pneumonia in undernourished children in rural Gambia
Source: Pneumonia (Nathan). 2026 Mar 25;18:8. doi: 10.1186/s41479-025-00194-8 (PMC13014833; doi:10.1186/s41479-025-00194-8)
Supplement: Supplementary file 1 — Supplementary Material 1 [file 41479_2025_194_MOESM1_ESM.docx]

**SUPPLEMENTARY MATERIAL**

**Figure S1 – Criteria to assign participants to a good and poor prognosis**.

| Prognosis | Condition at Discharge: Well Not Admitted | | Duration of Admission (Days) | | Decreased Feeding During Hospitalization | | Death in Hospital or Within 30-days of Follo Up | | Sought Care or Were Readmitted After Discharge | |
| --- | --- | --- | --- | --- | --- | --- | --- | --- | --- | --- |
|  | Yes | No | ≤ 3 | ≥ 7 | Yes | No | Yes | No | Yes | No |
| Good |  |  |  |  |  |  |  |  |  |  |
| Poor |  |  |  |  |  |  |  |  |  |  |
|  |  |  |  |  |  |  |  |  |  |  |

*One of the conditions to meet a poor prognosis was death during or after admission. The second condition was to: be discharged well, not have died during or within 30 days from the first encounter, and to have met any of the other criteria (linked by “or”). To meet a good outcome, participants had to meet all the stated conditions (linked by “and”).

**Figure S2 – Pneumonia prognosis according to the Stunting and Wasting Classification**

**Table S1 – Participant’s characteristics by undernutrition (wasting or stunting) status*.**

| **Participant Characteristic** | **Undernutrition**¥ | | |
| --- | --- | --- | --- |
|  | **Mild**  **(N = 83)** | **Moderate or Severe**  **(N = 162)** | **P†** |
| **Demographic** | | | |
| Age (months), median (IQR) | 15 (10, 26) | 12 (6, 20) | 0.0036 |
| Sex (female), n (%) | 41 (49%) | 70 (43%) | 0.34 |
| **Clinical History and Examination on Admission** | | | |
| Oxygen saturation (%), mean ± SD | 96.1 ± 5.6 | 97.4 ± 4.2 | 0.08 |
| Respiratory rate,/min, median (IQR) | 55 (48, 64) | 55 (46, 63) | 0.36 |
| Axilliary Temp, °C, mean ± SD | 37.7 ± 1.2 | 37.6 ± 1.1 | 0.44 |
| Antibiotic use (within past week), n (%) | 16 (20%) | 34 (22%) | 0.71 |
| Lethargy n, (%) | 9 (11%) | 52 (32%) | 0.0003 |
| Unconsciousness, n (%) | 1 (1.2%) | 1 (0.7%) | 1* |
| Convulsions, n (%) | 4 (4.8%) | 5 (3.1%) | 0.49* |
| Neck stiffness, n (%) | 1 (1.2%) | 1 (0.6%) | 1* |
| Bulging fontanel, n (%) | 69 (82%) | 137 (86%) | 0.45* |
| Diarrhea, n (%) | 11 (13%) | 61 (37%) | < 0.0001 |
| Vomiting, n (%) | 6 (7.2%) | 21 (13%) | 0.17 |
| **X-ray Results** | | | |
| Other infiltrates, n (%) | 4 (5.3%) | 5 (3.4%) | 0.49* |
| Consolidation, n (%) | 15 (20%) | 20 (14%) | 0.21* |
| Pleural Effusion, n (%) | 1 (1.1%) | 1 (0.7%) | 1* |
| **Undernutrition and Anemia** | | | |
| Hemoglobin, mean ± SD | 10.7 ± 1.4 | 9.9 ± 1.9 | 0.0001 |
| Anemia, n (%) |  |  | 0.016* |
| None | 36 (44%) | 46 (29%) |  |
| Mild | 21 (26%) | 65 (40%) |  |
| Moderate | 25 (30%) | 65 (40%) |  |
| Severe | 0 (0%) | 9 (5.6%) |  |
| Wasting, n (%) |  |  | < 0.0001* |
| None | 26 (31%) | 22 (13%) |  |
| Mild | 57 (69%) | 13 (8%) |  |
| Moderate | 0 (0%) | 65 (40%) |  |
| Severe | 0 (0%) | 63 (39%) |  |
| Stunting, n (%) |  |  | < 0.0001* |
| None | 43 (52%) | 56 (34%) |  |
| Mild | 40 (48%) | 35 (21%) |  |
| Moderate | 0 (0%) | 46 (28%) |  |
| Severe | 0 (0%) | 26 (16%) |  |
| **Infection and Inflammation Associated Laboratory Markers** | | | |
| WBC count (10^9^/L) mean ± SD | 14 ± 6 | 15 ± 9 | 0.13 |
| Neutrophil count (10^9^/L), median (IQR) | 6.4 (4.6, 10.6) | 6.0 (3.9, 10.0) | 0.44 |
| Neutrophil %, median (IQR) | 54 (42, 68) | 49 (35, 67) | 0.33 |
| Lymphocyte count (10^9^/L, median (IQR) | 4.60 (3.03, 6.30) | 4.62 (3.27, 6.40) | 0.61 |
| Lymphocyte %, median (IQR) | 37 (25, 51) | 40 (24, 53) | 0.63 |
| Hematocrit g/dL median (IQR) | 30 (28,33) | 30 (26,32) | 0.11 |
| Invasive Bacterial Infection, n (%) | 3 (3.6%) | 10 (6.1%) | 0.55 |
| Malarial Infection*, n (%) | 4 (4.9%) | 5 (3.1%) | 0.49 |

SD = standard deviation; WBC = white blood cells;

† P values were estimated with t tests when reporting means, Wilcoxon tests when reporting medians, and Pearson chi-square tests when reporting proportions except where marked * when Fisher exact tests were used.

¥ Undernutrition is defined as wasting (WHZ ≤ **-1)** or stunting (HAZ **≤ -1)**

**Table S2 – Characteristics of 198 participants with wasting (WHZ ≤ -1) by prognosis status.**

| **Participant Characteristic** | **Wasting** | | |
| --- | --- | --- | --- |
|  | **Good prognosis**  **(N = 62)** | **Poor prognosis**  **(N = 136)** | **P†** |
| **Demographic** | | | |
| Age (months), median (IQR) | 15 (9, 27) | 12 (6, 20) | 0.01 |
| Sex (female), n (%) | 32 (52%) | 61 (45%) | 0.38 |
| **Clinical History and Examination on Admission** | | | |
| Oxygen saturation (%), mean ± SD | 98.0 ± 2.3 | 96.1 ± 5.7 | 0.001 |
| Respiratory rate,/min, median (IQR) | 53 (48, 61) | 55 (46, 64) | 0.12 |
| Axilliary Temp, °C, mean ± SD | 37.68 ±1.15 | 37.65 ± 1.12 | 0.85 |
| Antibiotic use (within past week), n (%) | 11 (19%) | 28 (22%) | 0.67 |
| Lethargy n, (%) | 9 (15%) | 46 (34%) | 0.005 |
| Unconsciousness, n (%) | 0 (0%) | 1 (0.8%) | 1* |
| Convulsions, n (%) | 1 (1.6%) | 5 (3.7%) | 0.67* |
| Neck stiffness, n (%) | 0 (0%) | 1 (0.7%) | 1* |
| Bulging fontanel, n (%) | 50 (82%) | 113 (84%) | 0.68 |
| Diarrhea, n (%) | 16 (26%) | 51 (38%) | 0.11 |
| Vomiting, n (%) | 3 (4.8%) | 20 (15%) | 0.04* |
| **X-ray Results** | | | |
| Other infiltrates, n (%) | 1 (1.8%) | 7 (5.8%) | 0.44* |
| Consolidation, n (%) | 3 (5.3%) | 23 (19%) | 0.02* |
| Pleural Effusion, n (%) | 1 (1.8%) | 1 (0.8%) | 0.54* |
| **Undernutrition and Anemia** | | | |
| Hemoglobin, mean ± SD | 10.58 ± 1.57 | 9.93 ± 1.84 | 0.012 |
| Anemia, n (%) |  |  | 0.049* |
| None | 26 (42%) | 41 (30%) |  |
| Mild | 19 (31%) | 29 (21%) |  |
| Moderate | 16 (26%) | 58 (43%) |  |
| Severe | 1 (1.6%) | 7 (5.2%) |  |
| Weight-for-height Z-score, mean ± SD | -2.17 ± 0.86 | -2.92 ± 1.42 | < 0.0001 |
| Height-for-age Z-score, mean ± SD | -0.38 ± 1.52 | -1.17 ± 1.66 | 0.001 |
| Wasting, n (%) |  |  | 0.003 |
| Mild | 30 (48%) | 40 (29%) |  |
| Moderate | 22 (35%) | 43 (32%) |  |
| Severe | 10 (16%) | 53 (39%) |  |
| Stunting, n (%) |  |  | 0.001* |
| None | 43 (69%) | 56 (41%) |  |
| Mild | 11 (18%) | 38 (28%) |  |
| Moderate | 7 (11%) | 25 (18%) |  |
| Severe | 1 (1.6%) | 17 (13%) |  |
| **Infection and Inflammation Associated Laboratory Markers** | | | |
| WBC count (10^9^/L) mean ± SD | 14 ± 7 | 16 ± 9 | 0.2 |
| Neutrophil count (10^9^/L), median (IQR) | 5.6 (3.4, 10.1) | 6.4 (4.0, 10.0) | 0.49 |
| Neutrophil %, median (IQR) | 50 (38, 65) | 52 (37, 70) | 0.69 |
| Lymphocyte count (10^9^/L, median (IQR) | 4.66 (3.05, 6.37) | 4.60 (3.18, 6.37) | 0.76 |
| Lymphocyte %, median (IQR) | 39 (24, 53) | 38 (25, 52) | 0.81 |
| Hematocrit g/dL median (IQR) | 30.2 (28.3, 32.6) | 29.4 (26.3, 32.3) | 0.09 |
| Invasive Bacterial Infection, n (%) | 2 (3.2%) | 11 (8.1%) | 0.35* |
| Malarial Infection*, n (%) | 2 (3.2%) | 6 (4.5%) | 1* |

SD = standard deviation; WBC = white blood cells;

† P values were estimated with t tests when reporting means, Wilcoxon tests when reporting medians, and Pearson chi-square tests when reporting proportions except where marked * when Fisher exact tests were used.

**Table S3 - Characteristics of 147 participants with stunting (HAZ ≤ -1) by prognosis status.**

| **Participant Characteristic** | **Stunting** | | |
| --- | --- | --- | --- |
|  | **Good prognosis**  **(N = 38)** | **Poor prognosis**  **(N = 109)** | **P†** |
| **Demographic** | | | |
| Age (months), median (IQR) | 15 (9, 27) | 12 (6, 20) | 0.01 |
| Sex (female), n (%) | 13 (34%) | 47 (43%) | 0.34 |
| **Clinical History and Examination on Admission** | | | |
| Oxygen saturation (%), mean ± SD | 97.8 ± 2.78 | 96.8 ± 5.2 | 0.14 |
| Respiratory rate,/min, median (IQR) | 53 (48, 61) | 55 (46, 64) | 0.12 |
| Axilliary Temp, °C, mean ± SD | 37.37 ± 1.13 | 37.49 ± 1.23 | 0.56 |
| Antibiotic use (within past week), n (%) | 5 (14%) | 21 (21%) | 0.40 |
| Lethargy n, (%) | 5 (13%) | 34 (31%) | 0.03 |
| Unconsciousness, n (%) | 0 (0%) | 1 (1.0%) | 1* |
| Convulsions, n (%) | 1 (2.6%) | 5 (4.6%) | 1* |
| Neck stiffness, n (%) | 0 (0%) | 2 (1.8%) | 1* |
| Bulging fontanel, n (%) | 34 (89%) | 87 (81%) | 0.21 |
| Diarrhea, n (%) | 7 (18%) | 42 (39%) | 0.02 |
| Vomiting, n (%) | 2 (5.3%) | 17 (16%) | 0.16* |
| **X-ray Results** | | | |
| Other infiltrates, n (%) | 1 (2.9%) | 3 (3.0%) | 1* |
| Consolidation, n (%) | 1 (2.9%) | 26 (26%) | 0.003* |
| Pleural Effusion, n (%) | 0 (0%) | 1 (1.0%) | 1* |
| **Undernutrition and Anemia** | | | |
| Hemoglobin, mean ± SD | 10.75 ± 1.71 | 9.73 ± 1.98 | 0.004 |
| Anemia, n (%) |  |  | 0.005* |
| None | 18 (49%) | 28 (26%) |  |
| Mild | 11 (30%) | 21 (19%) |  |
| Moderate | 7 (19%) | 52 (48%) |  |
| Severe | 1 (2.7%) | 7 (6.5%) |  |
| Weight-for-height Z-score, mean ± SD | -1.07 ± 1.48 | -2.15 ± 2.09 | 0.0008 |
| Height-for-age Z-score, mean ± SD | -1.95 ± 0.75 | -2.25 ± 1.13 | 0.06 |
| Stunting, n (%) |  |  | 0.06 |
| Mild | 22 (58%) | 53 (49%) |  |
| Moderate | 14 (37%) | 32 (29%) |  |
| Severe | 2 (5.3%) | 24 (22%) |  |
| Wasting, n (%) |  |  | 0.01* |
| None | 19 (50%) | 29 (27%) |  |
| Mild | 7 (18%) | 20 (18%) |  |
| Moderate | 8 (21%) | 21 (19%) |  |
| Severe | 4 (11%) | 39 (36%) |  |
| **Infection and Inflammation Associated Laboratory Markers** | | | |
| WBC count (10^9^/L) mean ± SD | 13 ± 7 | 16 ± 9 | 0.13 |
| Neutrophil count (10^9^/L), median (IQR) | 5.6 (3.4, 10.1) | 6.4 (4.0, 10.0) | 0.49 |
| Neutrophil %, median (IQR) | 50 (38, 65) | 52 (37, 70) | 0.69 |
| Lymphocyte count (10^9^/L, median (IQR) | 4.66 (3.05, 6.37) | 4.60 (3.18, 6.37) | 0.76 |
| Lymphocyte %, median (IQR) | 39 (24, 53) | 38 (25, 52) | 0.81 |
| Hematocrit g/dL median (IQR) | 30.0 (5.6) | 28.7 (5.6) | 0.21 |
| Invasive Bacterial Infection, n (%) | 1 (2.6%) | 7 (6.4%) | 0.68* |
| Malarial Infection*, n (%) | 1 (2.6%) | 3 (2.8%) | 1* |

SD = standard deviation; WBC = white blood cells

† P values were estimated with t tests when reporting means, Wilcoxon tests when reporting medians, and Pearson chi-square tests when reporting proportions except where marked * when Fisher exact tests were used.

**Figure S3** - **Prognostic signatures considering clinical parameters other than Z-scores and including (A) and excluding (B) parameters from a full blood cell count among candidate predictors.** Ellipses contain name of variables discriminating poor from good prognosis in intermediate nodes. Terminal nodes (squares) include the predicted prognosis of participants, the number (and percent %) of participants in the node, as well as the proportion of participants in each node with a poor prognosis.

**
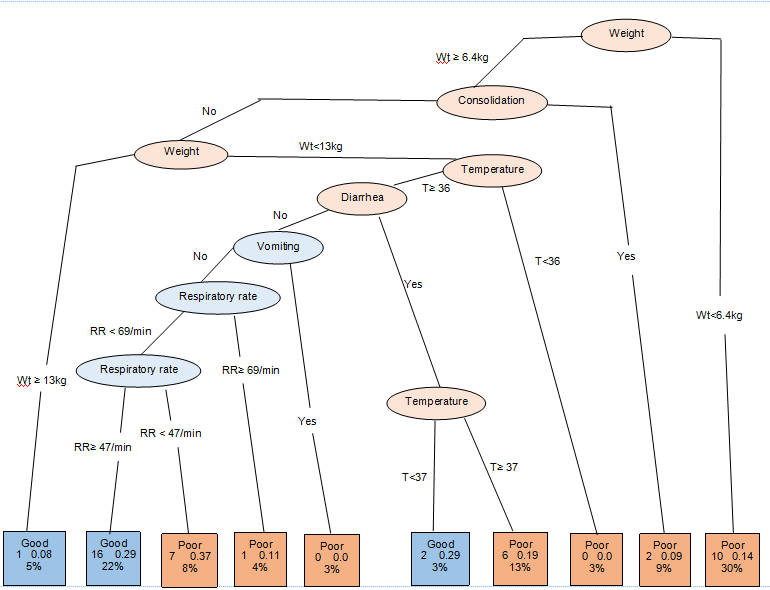
**

**A)**

**B)**


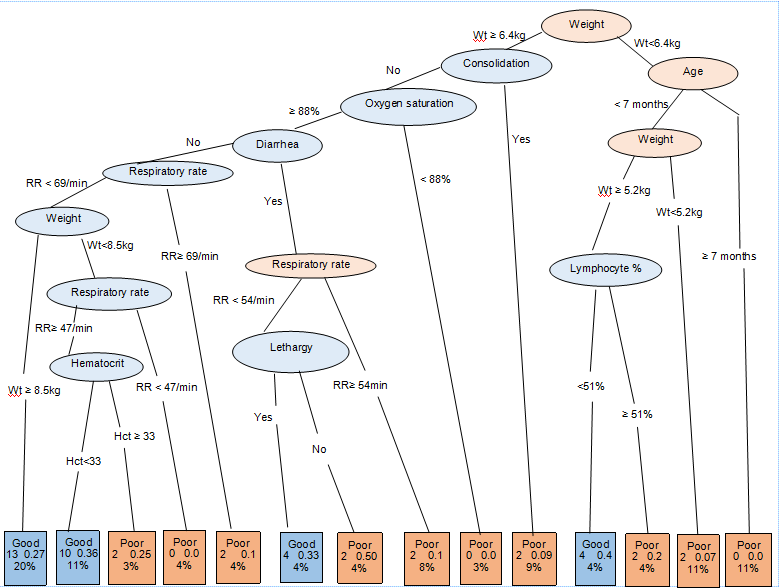


< 6.1 kg

< 54/min

≥ 54/min

< 19X10^9^/L

≥ 19X10^9^/L

**Table S4 – Accuracy of prognostic signatures identified through classification tree (Figure s2) and logistic penalized regression (fit through elastic net; Table S4): sensitivity, specificity, overall accuracy and AUC-ROC, all with corresponding 95% confidence intervals (CIs).**

|  | **Sensitivity**  **(95% CI)** | **Specificity**  **(95% CI)** | **Overall Accuracy**  **(95% CI)** | **AUC-ROC** |
| --- | --- | --- | --- | --- |
|  | **ALL PARTICIPANTS WITH UNDERNUTRITION** | | | |
| Tree with Clinical Predictors without Z-scores | 88% (85%, 90%) | 68% (57%, 78%) | 0.82 (0.76, 0.86) | 0.84 (0.78, 0.89) |
| Tree with Clinical + Lab Predictors without Z-scores | 82% (75%, 87%) | 83% (73%, 90%) | 0.82 (0.77, 0.87) | 0.87 (0.82, 0.91) |
| ENET with Clinical + Lab Predictors without Z-scores | 83% (74%, 90%) | 51% (37%, 65%) | 0.72 (0.65, 0.79) | 0.75 (0.67, 0.83) |

**Figure S4 -- Prognostic signature among 198 participants with wasting (weight-for-height Z-score ≤ -1) obtained in classification trees:** In (A), Z-scores and full blood cell count (FBC) parameters were included among the pool of candidate predictors when fitting the tree; in (B) Z-scores were included BUT FBC parameters were excluded from the pool of candidate predictors; in (C) Z-scores were excluded BUT FBC parameters were included in the pool of candidate predictors; in (D) both full blood cell count and FBC parameters were excluded from the pool of candidate predictors. Predictors that split the threes at each level are shown below intermediate nodes. Terminal nodes show the final predicted category of each combination of predictors. Numbers in all nodes represent the proportion of participants with a poor prognosis and the percentage of the total participants that are included in the nodes.

**(A)**


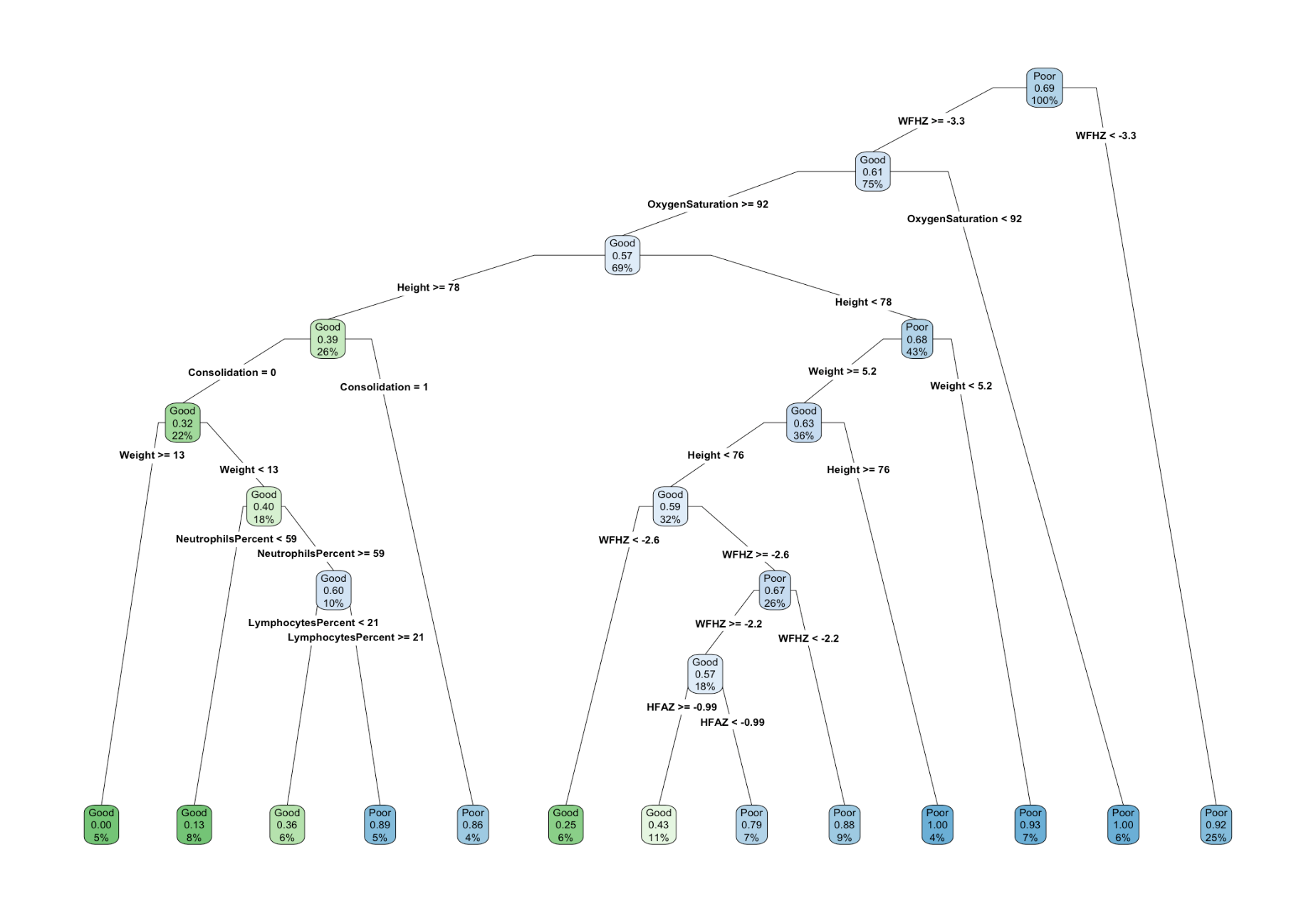


WFHZ: Weight for height z-score HFAZ: Height for age z-score


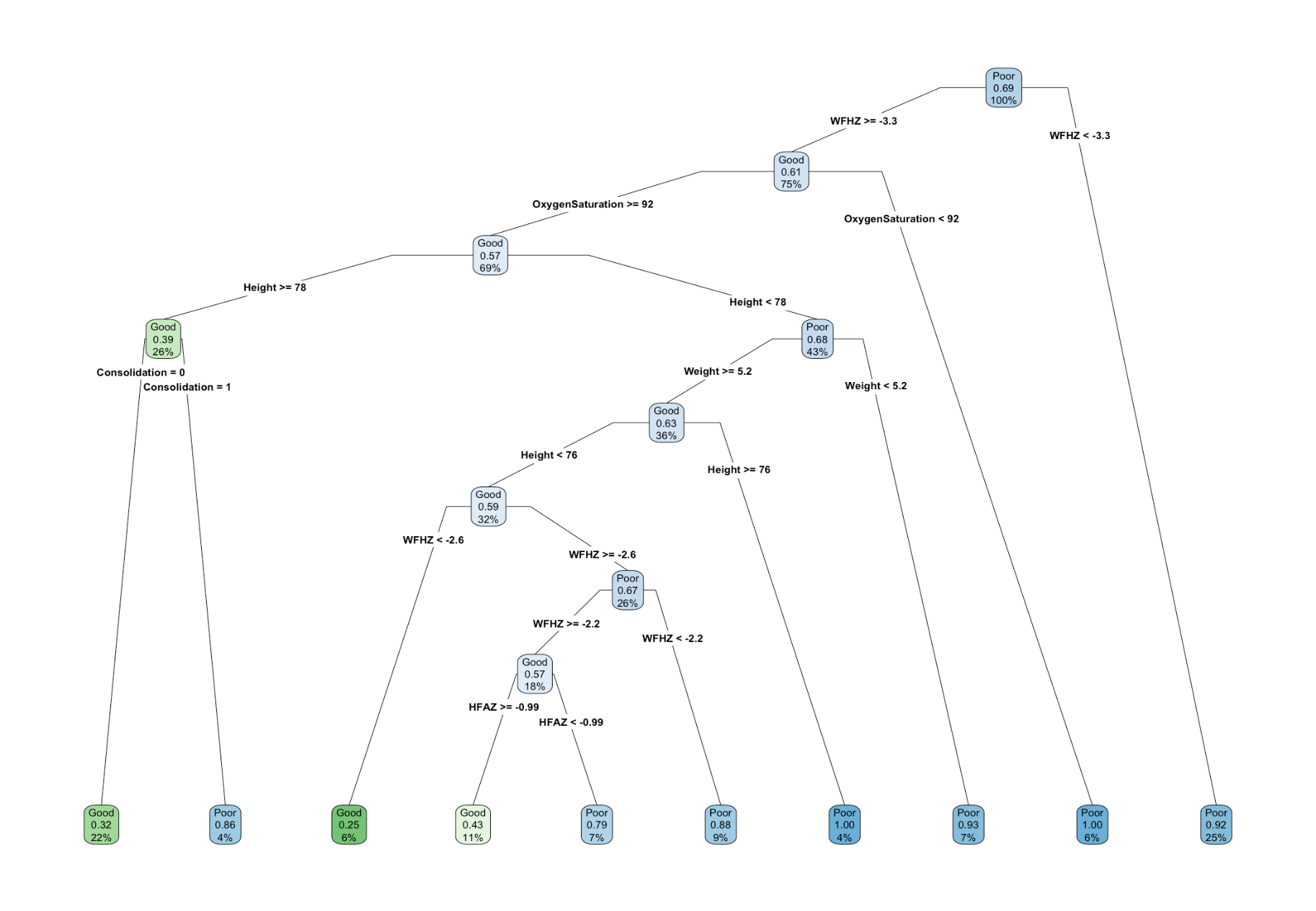
**(B)**

WFHZ: Weight for height z-score HFAZ: Height for age z-score


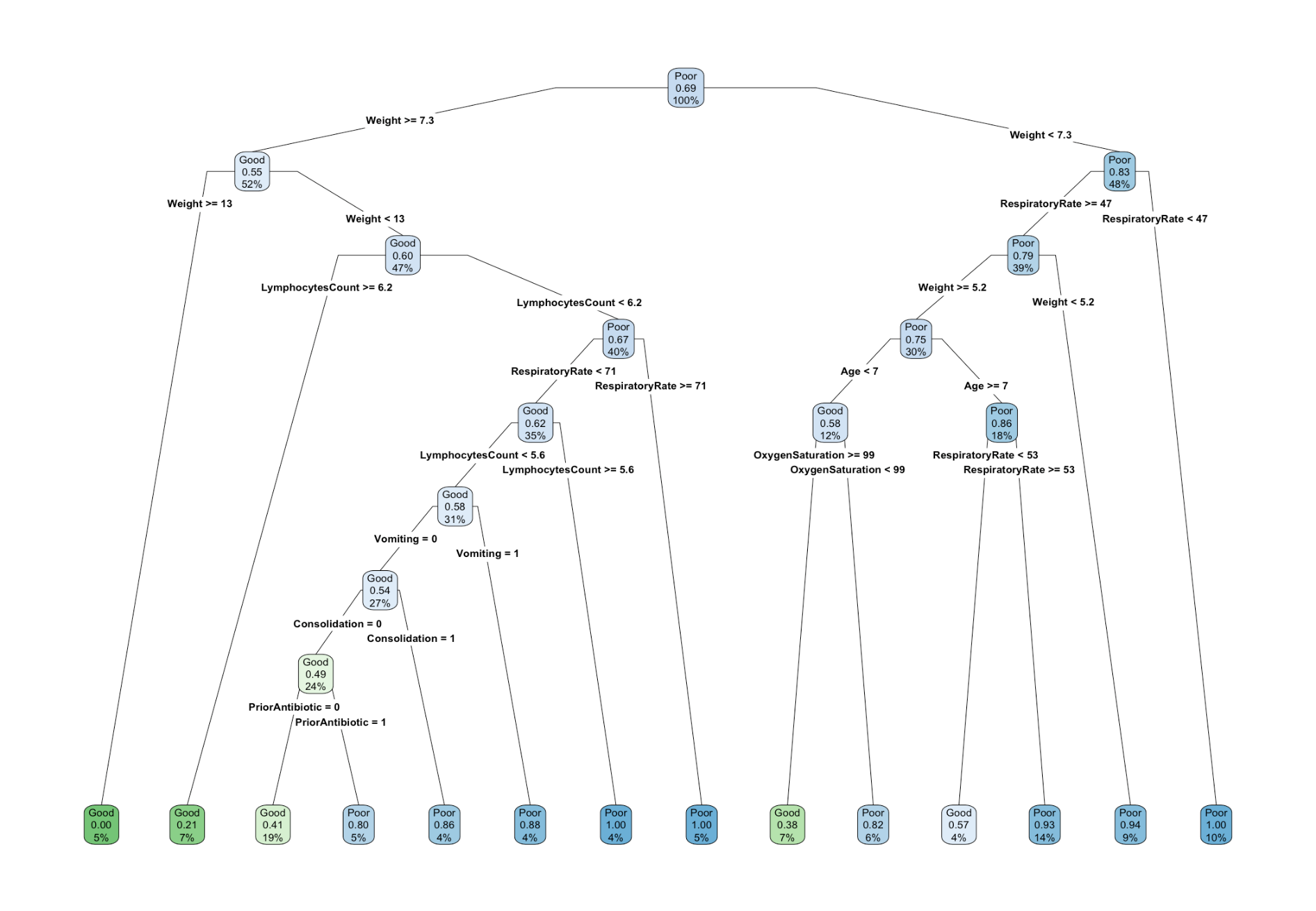
**(C)**


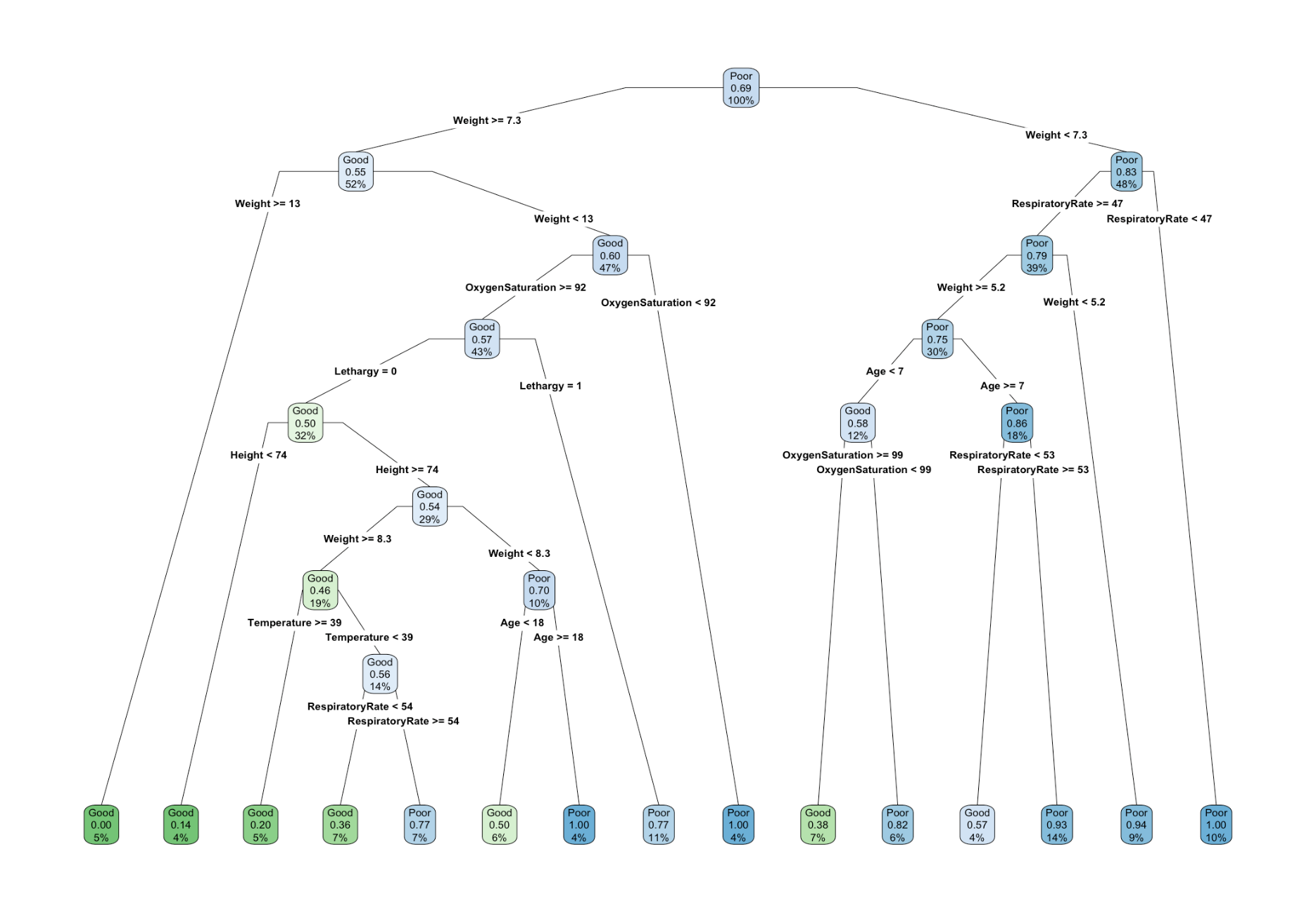
**(D)**

**Figure S5 - Prognostic signature among 147 participants with stunting (height-for-age Z-score ≤ -1) obtained in classification trees including (A) and excluding (B) parameters from a full blood cell count among candidate predictors.** In (A), Z-scores and full blood cell count (FBC) parameters were included among the pool of candidate predictors when fitting the tree; in (B) Z-scores were included BUT FBC parameters were excluded from the pool of candidate predictors; in (C) Z-scores were excluded BUT FBC parameters were included in the pool of candidate predictors; in (D) both full blood cell count and FBC parameters were excluded from the pool of candidate predictors. Predictors that split the threes at each level are shown below intermediate nodes. Terminal nodes show the final predicted category of each combination of predictors. Numbers in all nodes represent the proportion of participants with a poor prognosis and the percentage of the total participants that are included in the nodes.


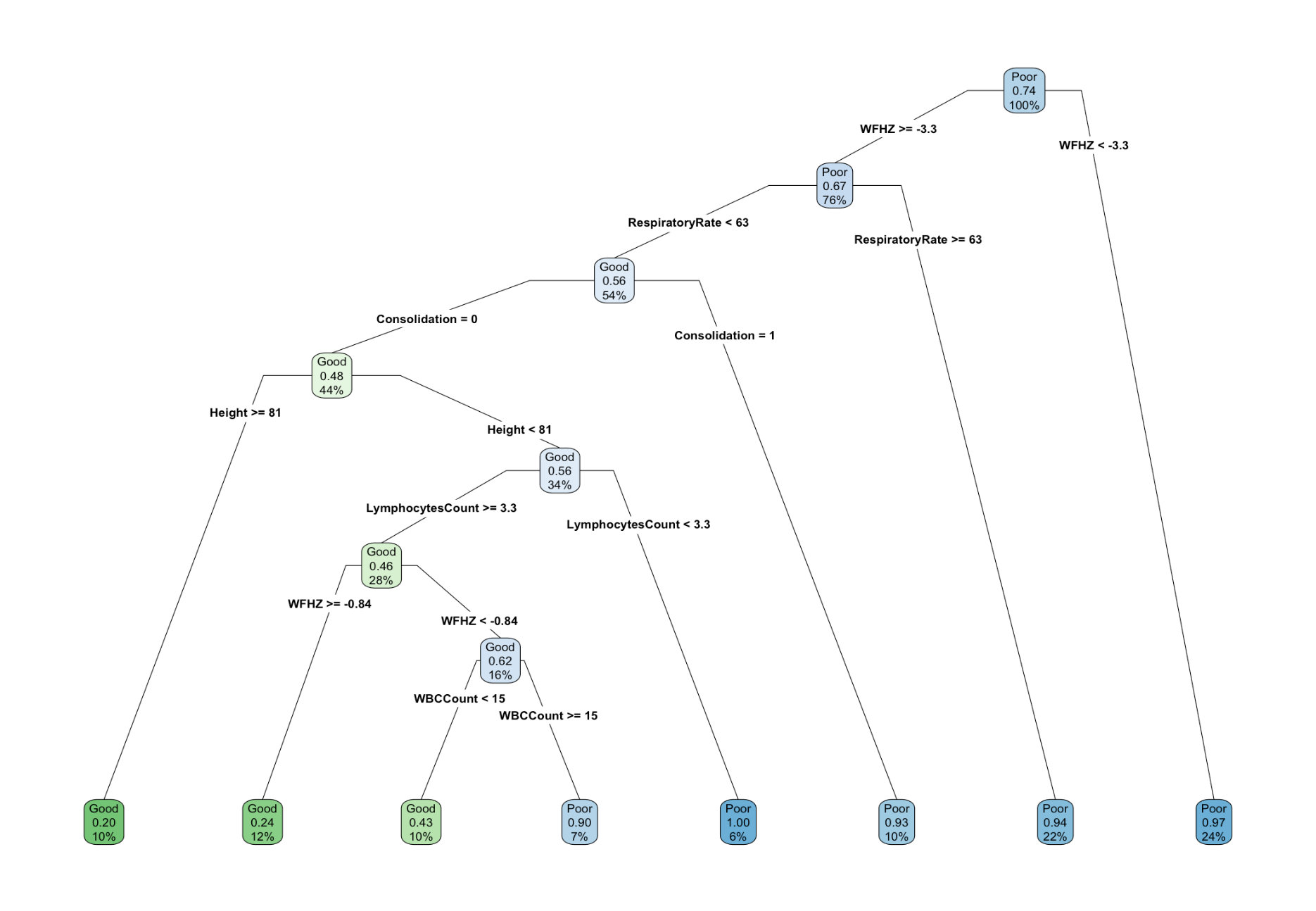
**(A)**

WFHZ: Weight for height z-score WBC: White blood cell count


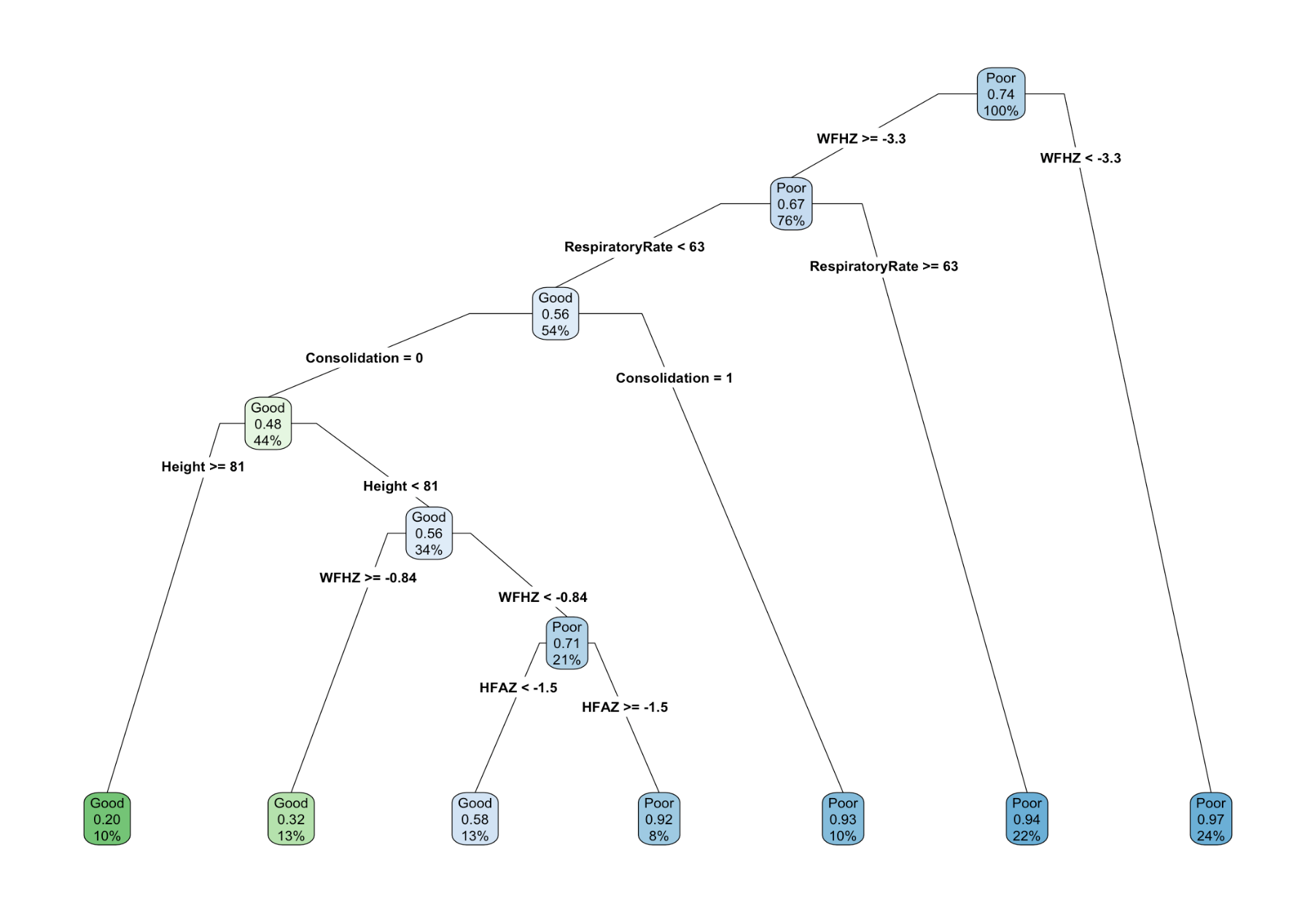
**(B)**

WFHZ: Weight for height z-score HFAZ: Height for age z-score


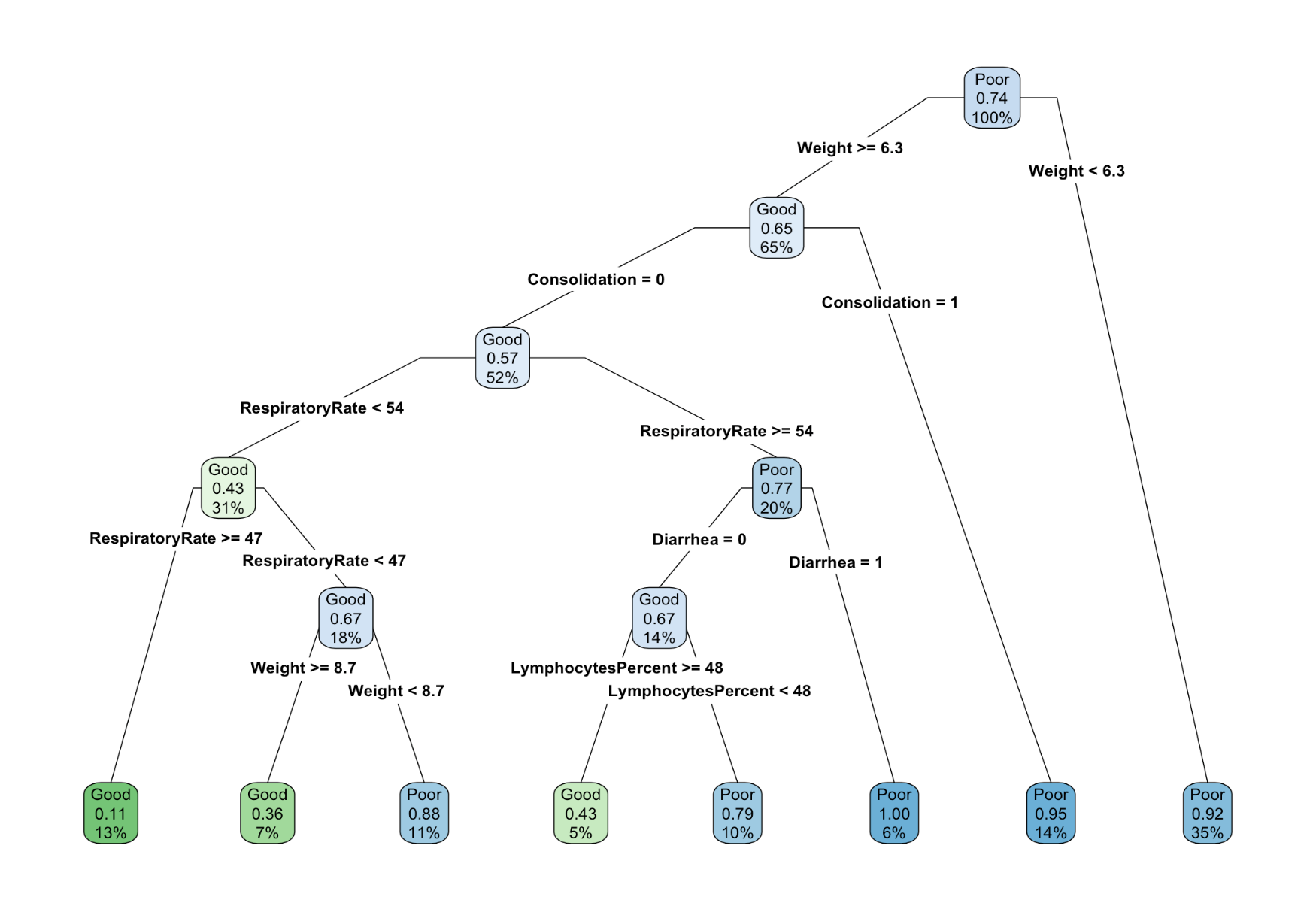
**(C)**


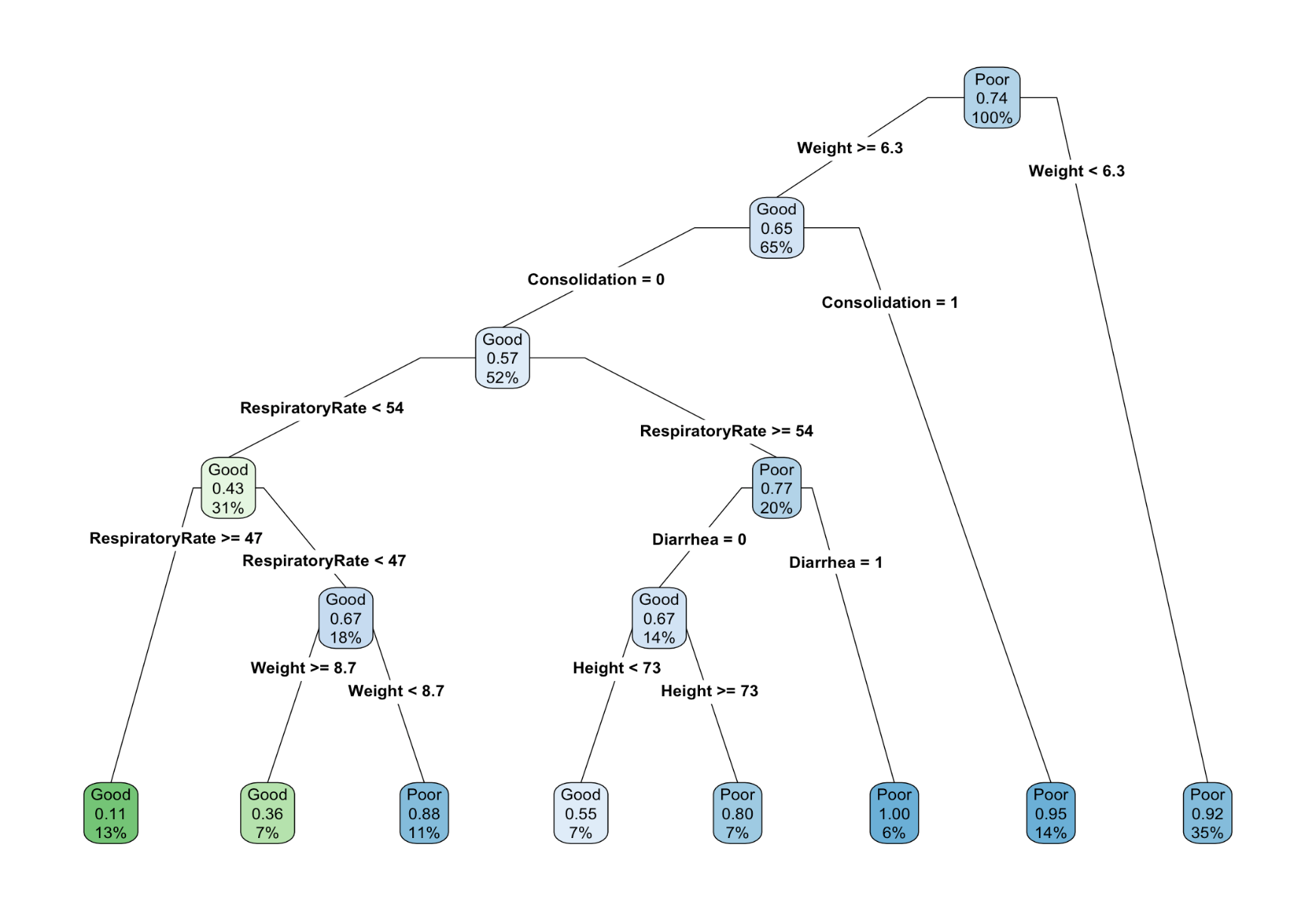
**(D)**

**Table S5 - Accuracy of all prognostic signatures in children with wasting and stunting. Signatures based on classification trees included 198 participants with wasting (62 with good and 136 with poor prognosis) and 147 with stunting (38 with good and 109 with poor prognosis). Elastic net (ENET) signatures included 125 participants with wasting (40 with good and 85 with poor prognosis) and 94 with stunting (25 with good and 69 with poor prognosis).**

|  | **Sensitivity**  **(95% CI)** | **Specificity**  **(95% CI)** | **Overall Accuracy**  **(95% CI)** | **AUC-ROC** |
| --- | --- | --- | --- | --- |
|  | **PARTICIPANTS WITH WASTING** | | | |
| Tree with Clinical Predictors | 83% (76%, 89%) | 79% (67%, 88%) | 0.82 (0.76, 0.87) | 0.89 (0.84, 0.93) |
| Tree with Clinical + Lab Predictors | 80% (73%, 87%) | 85% (77%, 94%) | 0.82 (0.76, 0.87) | 0.89 (0.84, 0.93) |
| Tree with Clinical Predictors + Z-scores | 87% (80%, 92%) | 81% (69%, 90%) | 0.85 (0.79, 0.90) | 0.85 (0.84, 0.94) |
| Tree with Clinical + Lab Predictors + Z-scores | 81% (73%, 87%) | 82% (70%, 91%) | 0.81 (0.75, 0.86) | 0.89 (0.84, 0.93) |
| ENET with Clinical + Lab Predictors | 89% (81%, 95%) | 40% (25%, 57%) | 0.74 (0.65, 0.81) | 0.72 (0.62, 0.81) |
| ENET with Clinical + Lab Predictors + Z-scores | 89% (81%, 95%) | 35% (21%, 52%) | 0.72 (0.63, 0.80) | 0.73 (0.64, 0.82) |
|  | **PARTICIPANTS WITH STUNTING** | | | |
| Tree with Clinical Predictors | 92% (85%, 96%) | 74% (57%, 87%) | 0.87 (0.81, 0.92) | 0.88 (0.81, 0.95) |
| Tree with Clinical + Lab Predictors | 89% (82%, 94%) | 76% (60%, 89%) | 0.86 (0.79, 0.91) | 0.88 (0.81, 0.95) |
| Tree with Clinical Predictors + Z-scores | 82% (73%, 88%) | 87% (72%, 96%) | 0.83 (0.76, 0.89) | 0.91 (0.85, 0.96) |
| Tree with Clinical + Lab Predictors + Z-scores | 88% (80%, 93%) | 87% (72%, 96%) | 0.88 (0.81, 0.93) | 0.88 (0.82, 0.95) |
| ENET with Clinical + Lab Predictors | 90% (80%, 96%) | 48% (28%, 69%) | 0.79 (0.69, 0.86) | 0.80 (0.70, 0.89) |
| ENET with Clinical + Lab Predictors + Z-scores | 90% (80%, 96%) | 48% (28%, 69%) | 0.79 (0.69, 0.86) | 0.80 (0.70, 0.89) |

**Table S6 – Prognostic signature for 246 children with mild to severe undernutrition (WHZ or HAZ ≤ -1), comparing those with poor (N = 165 children) and good (N = 81) prognoses estimated through a penalized logistic regression model (fit through Elastic Net).** Signatures included parameters of full blood cell count among candidate predictors but no Z-scores and only participants with full blood cell count were included in this analysis.

| **Predictors** | **Odds Ratios** |
| --- | --- |
| ***Not including Z-scores among candidate predictors*** | |
| Intercept | 2.19 |
| Lethargy (yes) | 1.02 |
| Diarrhea (yes) | 0.09 |
| Vomit (yes) | 1.14 |
| Consolidation in the chest X-ray | 2.27 |
| Weight (Kg) | 0.91 |

**Table S7 - Prognostic signature for 125 participants with wasting (weight-for-height Z-score ≤ -2) comparing those with good (N = 40 participants) and poor (N = 85 participants) prognoses estimated through a logistic regression model fit through elastic net.** Candidate predictors included clinical and laboratory markers. Participants with missing full blood cell count were excluded from this analysis.

| **Predictors** | **Odds Ratio** |
| --- | --- |
| ***Not including Z-scores among candidate predictors*** | |
| Intercept | 0.27 |
| O_2_ Saturation | 0.99 |
| Diarrhea (yes) | 1.43 |
| Weight (Kg) | 0.87 |
| ***Including Z-scores among candidate predictors*** | |
| Intercept | 2.72 |
| Consolidation in the chest X-ray | 1.26 |
| Weight (Kg) | 1.13 |
| Weigh-for-height Z-scores | 0.97 |
| Height-for-age Z-scores | 1.05 |

**Table S8 - Prognostic signature for 94 participants with stunting (height-for-age Z-score ≤ -2) comparing those with good (N = 25 participants) and poor (N = 69 participants) prognoses estimated through a logistic regression model fit through elastic net.** Candidate predictors included clinical and laboratory markers. Participants with missing full blood cell count were excluded from this analysis.

| **Predictors** | **Odds Ratio** |
| --- | --- |
| ***Not including Z-scores among candidate predictors*** | |
| Intercept | 1.46 |
| Diarrhea (yes) | 1.09 |
| Respiratory rate | 1.002 |
| Consolidation in the chest X-ray | 1.73 |
| Weight (Kg) | 0.94 |
| ***Including Z-scores among candidate predictors**** | |
| Intercept | 1.46 |
| Diarrhea (yes) | 1.09 |
| Respiratory rate | 1.002 |
| Consolidation in the chest X-ray | 1.73 |
| Weight (Kg) | 0.94 |

* The same model was selected when including or excluding Z-scores among candidate predictors
